# Targeting PTP4A3 with KVX-053 mitigates alcohol-amplified SARS-CoV-2 spike protein–induced acute lung injury

**DOI:** 10.3389/fphar.2026.1862783

**Published:** 2026-06-19

**Authors:** Pavel A. Solopov, Ruben Manuel Luciano Colunga Biancatelli, Elizabeth R. Sharlow, John S. Lazo, John D. Catravas

**Affiliations:** 1 Frank Reidy Research Center for Bioelectrics, Old Dominion University, Norfolk, VA, United States; 2 Division of Pulmonary and Critical Care, Department of Internal Medicine, Eastern Virginia Medical School, Macon & Joan Brock Virginia Health Sciences at Old Dominion University, Norfolk, VA, United States; 3 Department of Pharmacology, School of Medicine, University of Virginia, Charlottesville, VA, United States; 4 KeViRx, Inc., Charlottesville, VA, United States; 5 School of Medical Diagnostic & Translational Sciences, Ellmer College of Health Sciences, Old Dominion University, Norfolk, VA, United States

**Keywords:** alcohol, ARDS, aud, KVX-053, lung injury, PTP4A3

## Abstract

**Introduction:**

Chronic alcohol exposure is associated with increased vulnerability to acute respiratory distress syndrome (ARDS), and emerging evidence suggests that this susceptibility extends to the severe respiratory manifestations of COVID- 19. The SARS-CoV-2 Spike Protein S1 subunit (S1SP) can independently trigger robust inflammatory and vascular injury responses in the lung, and our prior work demonstrated that chronic alcohol consumption markedly intensifies these effects in mice. PTP4A3 phosphatase is a key regulator of inflammation and endothelial barrier function. In this study, we investigated whether the PTP4A3 inhibitor KVX-053 can counteract alcohol-enhanced S1SP-induced lung injury in K18-hACE2 mice.

**Methods:**

Animals were maintained on control or chronic ethanol diets, intratracheally exposed to S1SP, and treated with KVX-053 once a day for the next 3 days.

**Results:**

Seventy-two hours after S1SP exposure, ethanol-fed mice exhibited significantly greater leukocyte accumulation and protein leakage in bronchoalveolar lavage fluid, alongside heightened production of IL-6, TNF-α, and TGF-β. These amplified responses were accompanied by increased activation of NF-κB, STAT3, and the NLRP3 inflammasome, as well as elevated ACE2 expression.

**Conclusions:**

KVX-053 markedly blunted each of these alcohol-dependent injury parameters, reducing cytokine levels, normalizing barrier permeability, and suppressing downstream inflammatory signaling.

## 1 Introduction

Acute respiratory distress syndrome (ARDS) is one of the primary causes of death in patients with severe COVID-19 (Cheyne et al., 2023). The syndrome is characterized by diffuse inflammation, endothelial injury, and loss of alveolar–capillary barrier integrity (Flaumenhaft et al., 2022). Increasing evidence indicates that these pathological features are not driven solely by viral replication. Instead, host inflammatory responses activated by the SARS-CoV-2 Spike protein, particularly its S1 subunit, play a major role in the progression of lung injury (Buzhdygan et al., 2020; Colunga Biancatelli et al., 2021; Dorw et al., 2021; Lei et al., 2021).

Several groups have demonstrated that the S1 subunit of Spike Protein (S1SP) can independently activate inflammatory signaling, disrupt endothelial barrier function, and induce vascular leakage in the lung even in the absence of infectious virus (Lei et al., 2021; Rysz et al., 2021). Our previous work similarly showed that intratracheal S1SP instillation in K18-hACE2 mice recapitulates key features of COVID-19–related lung injury, including cytokine elevation, alveolar protein leakage, and STAT3/NF-κB activation outside of an infectious context (Colunga Biancatelli et al., 2021). Rare clinical reports have described intense inflammatory reactions following mRNA vaccination in the absence of detectable virus, suggesting that Spike protein expressed from mRNA platforms, under uncommon circumstances, may elicit tissue-level inflammatory responses similar to those seen during infection (Kim et al., 2021; Shay et al., 2021; Yoshimura et al., 2022). Circulating S1 fragments have been detected transiently in vaccinated individuals, reinforcing the concept that Spike protein alone can exert measurable biological effects (Ogata et al., 2022). Although these events are rare compared with the overwhelming safety and benefit of vaccination, they support the broader mechanistic understanding that Spike protein, independent of live virus, can activate inflammatory pathways relevant to COVID-19 induced ARDS.

Chronic alcohol consumption introduces an additional risk factor that substantially modifies pulmonary immune responses. Individuals with long-term alcohol use disorder (AUD) display impaired mucociliary clearance, reduced alveolar macrophage function, increased oxidative stress, and weakened epithelial and endothelial barriers, all of which predispose the lung to enhanced inflammatory injury during respiratory infections (Mehta and Guidot, 2017; Simet and Sisson, 2015). Clinically, these abnormalities are associated with higher rates of bacterial pneumonia, sepsis, and ARDS in people with AUD. During the COVID-19 pandemic, large-scale electronic-health-record analyses and cohort studies showed that patients with documented AUD or unhealthy alcohol use had higher odds of hospitalization, severe disease, and all-cause mortality after SARS-CoV-2 infection compared with individuals without AUD (Bailey A. J. et al., 2022; Bhalla et al., 2021).

Experimentally, the interaction between alcohol and Spike-mediated lung injury has been explored in detail. In K18-hACE2 transgenic mice, chronic ethanol feeding increases lung ACE2 expression, amplifies S1SP-induced cytokine production, worsens vascular leakage and histologic lung damage, and enhances activation of STAT3, NF-κB, and NLRP3 inflammasome pathways (Solopov et al., 2022a). Independent work in rodent models has shown that chronic alcohol intake upregulates ACE2 and related renin–angiotensin pathway genes in the lung and other organs, thereby increasing the theoretical probability of SARS-CoV-2 entry into target cells (Friske et al., 2023). Chronic alcohol use also modulates key inflammatory cascades: it alters NF-κB signaling in monocytes and other immune cells and dysregulates NF-κB/STAT3 pathways in parenchymal tissues, contributing to exaggerated cytokine responses to secondary insults (Jaruga et al., 2004; Mandrekar et al., 1999). Our recent review further summarized how alcohol-induced defects in dendritic cell function, T-cell responses, and cytokine regulation may potentiate Spike-driven inflammation during COVID-19 or in the context of Spike-based vaccination (Solopov, 2023).

PTP4A3 (also known as PRL-3) is a dual-specificity protein phosphatase implicated in the regulation of cytoskeletal organization, cellular stress responses, and pro-inflammatory signal transduction. Aberrant PTP4A3 expression has been shown to drive activation of key inflammatory transcriptional programs: PTP4A3 overexpression induces constitutive STAT3 phosphorylation and signaling in IL-6–dependent myeloma cells, whereas pharmacologic or genetic PTP4A3 inhibition reduces STAT3 activation (Rubio and Köhn, 2016; Slørdahl et al., 2016). In parallel, PTP4A3 directly enhances NF-κB signaling by interacting with RAP1 and increasing phosphorylation of the p65 NF-κB subunit, thereby promoting NF-κB–dependent gene expression (Rubio and Köhn, 2016; Lian et al., 2013). These STAT3 and NF-κB pathways are well-recognized mediators of cytokine amplification, endothelial dysfunction, and barrier breakdown in acute lung injury and ARDS, providing a mechanistic rationale for targeting PTP4A3 (Colunga Biancatelli et al., 2025a; Solopov et al., 2024).

Pharmacologic inhibition of PTP4A3 with the small-molecule inhibitor KVX-053 has been shown to counteract these responses by reducing STAT3 activation and dampening NF-κB signaling, resulting in decreased cytokine production and improved barrier function in endothelial and pulmonary injury models (Colunga Biancatelli et al., 2025a; Manuel Luciano Colunga Biancatelli et al., 2023). In SARS-CoV-2 infection models, KVX-053 administration attenuated lung inflammation, suppressed phosphorylation of STAT3, and lowered expression of NF-κB downstream targets, leading to a marked reduction in histologic lung injury (Colunga-Biancatelli et al., 2025a; Solopov et al., 2024).

Given this mechanistic profile, we hypothesized that KVX-053 would counteract the pathological inflammatory amplification observed when SARS-CoV-2 Spike protein signaling intersects with alcohol-induced immune dysregulation. Both STAT3 and NF-κB serve as critical convergence points for Spike-triggered cytokine production and alcohol-primed inflammatory sensitivity, and PTP4A3 inhibition directly attenuates these pathways. To address this hypothesis, we employed a K18-hACE2 mouse model of S1SP-induced lung injury with chronic ethanol exposure and evaluated the therapeutic efficacy of KVX-053. We assessed pulmonary inflammation, cytokine production, barrier dysfunction, histologic lung injury, and activation of key inflammatory signaling pathways, including NF-κB, STAT3, and NLRP3.

## 2 Materials and methods

### 2.1 Animals and experimental groups

All procedures were conducted in accordance with protocols approved by the Old Dominion University Institutional Animal Care and Use Committee (protocol #20-012) and followed guidelines of the American Physiological Society for animal research. K18-hACE2 transgenic mice (8–10 weeks old; Jackson Laboratories, Bar Harbor, ME) were acclimated for 5 days while receiving the Lieber–DeCarli ‘82 control liquid diet (Bio-Serv, Flemington, NJ). After acclimation, animals were randomly assigned to experimental groups and maintained either on the control liquid diet or on the ethanol-containing Lieber–DeCarli diet. Male mice were used to minimize variability associated with sex-dependent hormonal fluctuations, common for lung injury models, and to maintain consistency with our previously published studies using this model (Solopov et al., 2022a; Solopov et al., 2021).

To establish chronic alcohol exposure, mice were transitioned gradually to a 5%–6% (v/v) ethanol diet over a 7-day period, followed by exclusive ethanol diet feeding for an additional 14 days. Diet formulations provided 36% of total calories from ethanol or isocaloric maltodextrin (control diet), 35% from fat, 11% from carbohydrate, and 18% from protein. Animals consumed liquid diet *ad libitum* (20–30 mL/day). This protocol reliably produces blood alcohol concentrations of approximately 180 mg/dL by 10 days of feeding.

Mice were assigned to one of the following groups:

1. Control + Vehicle–control diet + intratracheal (i.t.) instillation of saline (2 μL/g body weight).
2. Control + S1SP–control diet + i.t. SARS-CoV-2 S1SP (400 ng/g; 2 μL/g body weight).
3. Control + S1SP + KVX-053 – control diet + i.t. SARS-CoV-2 S1SP + KVX-053 (10 mg/kg, i.p.) 1, 24, and 48 h after S1SP instillation.
4. Ethanol + Vehicle–ethanol diet + i.t. instillation of saline.
5. Ethanol + S1SP–ethanol diet + i.t. instillation of S1SP.
6. Ethanol + S1SP + KVX-053 - ethanol diet + i.t. instillation of S1SP + KVX-053 (10 mg/kg, i.p.) 1, 24, and 48 h after S1SP instillation.

**FIGURE 1:**
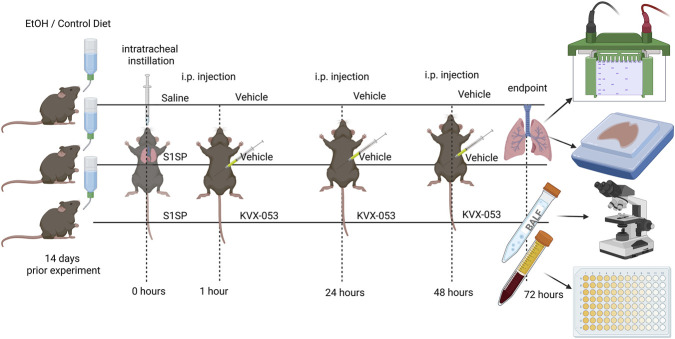
Experimental design of ethanol-enhanced S1SP-induced acute lung injury and KVX-053 treatment protocol. K18-hACE2 mice were maintained on either ethanol-containing or control liquid diets for 14 days before S1SP challenge. At time 0, mice received intratracheal instillation of SARS-CoV-2 Spike protein subunit 1 (S1SP) or saline vehicle. KVX-053 or vehicle was administered by intraperitoneal (i.p.) injection 1 h after S1SP instillation, followed by additional injections at 24 and 48 h. Animals were euthanized 72 h after S1SP administration. Bronchoalveolar lavage fluid (BALF), blood, and lung tissues were collected for analysis of inflammatory mediators, leukocyte counts, vascular permeability, histopathology, immunohistochemistry, and protein expression by Western blotting.

The experimental timeline is illustrated in Figure 1.

### 2.2 Intratracheal instillation of SARS-CoV-2 spike S1 subunit

Recombinant S1SP was prepared in sterile saline immediately before use. On day 19 of diet feeding, mice were anesthetized with ketamine (20–50 mg/kg)/xylazine (3–5 mg/kg) mixture, positioned supine, and a bolus of S1SP (400 μg/kg in saline; 2 mL/kg) was instilled into the trachea under direct visualization. Control mice received an equivalent volume of sterile saline. Following instillation, mice recovered fully before returning to their assigned diets.

### 2.3 KVX-053 preparation and administration

KVX-053 was dissolved in 30% Captisol, 40% PEG400, 30% PBS to ensure complete solubilization. Treatment began 1 h after S1SP instillation to model therapeutic post-injury intervention. The 1-h post-instillation time point was selected to model an early therapeutic intervention after lung injury initiation rather than prophylactic treatment, consistent with prior post-injury pharmacologic intervention strategies used in experimental lung injury models (Solopov et al., 2022b). KVX-053 was administered intraperitoneally (i.p.) once daily at a dose established in our preliminary studies to inhibit PTP4A3 activity *in vivo* (Colunga Biancatelli et al., 2022). Vehicle-treated animals received 100 µL of 30% Captisol, 40% PEG400, 30% PBS. All animals were monitored daily for health status and diet consumption. KVX-053 dosing mirrored the timeline and methodology indicated in the graphical experimental design (Figure 1).

### 2.4 Bronchoalveolar lavage (BALF) collection and cell counts

Seventy-two hours after S1SP administration, mice were euthanized with pentobarbital (100 mg/kg, i.p.), and the trachea was cannulated for bronchoalveolar lavage as described before (Solopov et al., 2020). Lungs were lavaged with 1 mL sterile PBS, which was withdrawn and centrifuged at 2,400 *g* for 10 min at 4 °C. Supernatant was aliquoted and stored at −80 °C for later biochemical assays. Cell pellets were resuspended in PBS and total leukocyte counts were determined using a hemocytometer, differential analysis was performed with the Wright-Giemsa stain kit.

### 2.5 Assessment of pulmonary vascular permeability

Total protein concentration in BALF was quantified using the micro-BCA assay (ThermoFisher Scientific, Waltham, MA). Protein levels were used as an index of alveolar–capillary barrier dysfunction.

### 2.6 Cytokine measurements in BALF

BALF concentrations of IL-6 (Cat # KMC0061), TNF-α, (Cat. # BMS607-3), TGF-β1 (Cat # BMS608-4), keratinocyte-derived chemokine (KC) (Cat. # EMCXCL1), and MCP-1 (Cat. # BMS281) were measured in triplicate using Invitrogen ELISA kits, obtained from ThermoFisher Scientific, according to manufacturer instructions. Cytokine concentrations were calculated from standard curves and normalized to BALF volume.

### 2.7 Histology, immunohistochemistry and lung injury scoring

Lungs were inflated and fixed via tracheal instillation of 10% neutral-buffered formalin at a pressure of 15 cm H_2_O, followed by immersion fixation for 72 h. Tissues were paraffin-embedded, sectioned at 5 μm, and stained with hematoxylin and eosin (H&E). Twenty non-overlapping fields per lung were examined under ×100 magnification by a blinded investigator. Lung injury was assessed using a modified scoring system evaluating neutrophil infiltration, hyaline membrane formation, alveolar wall thickening, and presence of proteinaceous debris (Matute-Bello et al., 2011). Immunostaining with PRL-3 (PTP4A3) antibody (Invitrogen, #PA5-96031) was performed on separate 5 μm sections, taken from the same paraffin blocks, as previously described (Solopov et al., 2024).

### 2.8 Lung tissue harvesting and protein extraction

After BALF collection, pulmonary circulation was flushed with PBS containing EDTA. Lungs were excised, snap-frozen in liquid nitrogen, and stored at −80 °C. Tissue was homogenized in RIPA buffer supplemented with protease and phosphatase inhibitors, followed by centrifugation at 14,000 × g for 10 min at 4 °C. Protein concentrations were measured by the BCA assay.

### 2.9 Western blotting

Equal amounts of total protein were mixed with tricine sample buffer, boiled for 5 min, resolved on 10% SDS-PAGE gels, and transferred to nitrocellulose membranes. Membranes were incubated with primary antibodies against phospho-STAT3 (Cell Signaling, #9145S), total STAT3 (#4904S), phospho-IκBα (#2859S), total IκBα (#9242S), NLRP3 (#15101), ACE2 (#4355), and β-actin (loading control, Invitrogen, #3290431), followed by IRDye-conjugated secondary antibodies. Blots were imaged using a LI-COR Odyssey CLx system. Densitometric quantification was performed using Fiji ImageJ v.1.53i.

### 2.10 Statistical analysis

Data are presented as mean ± SEM. Statistical analyses were performed using GraphPad Prism 8. Statistical comparisons were made using one-way or two-way ANOVA followed by Tukey’s *post hoc* test. A significance threshold of p < 0.05 was applied. Group sizes (n = 4–5) were consistent with our previous studies and sufficient to detect differences in established injury endpoints.

## 3 Results

### 3.1 Body weight changes during ethanol feeding and S1SP-induced lung injury

**FIGURE 2:**
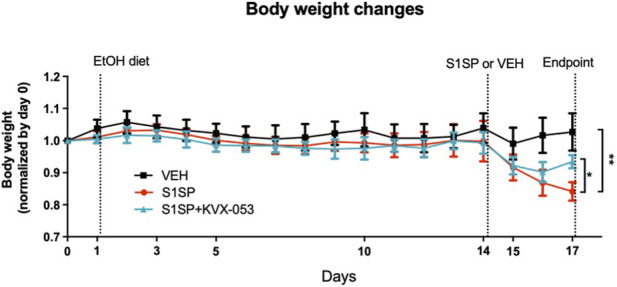
Body weight changes during ethanol feeding and S1SP-induced lung injury. Body weight was monitored daily in K18-hACE2 mice maintained on an EtOH diet and treated with vehicle (VEH), SARS-CoV-2 Spike protein subunit 1 (S1SP), or S1SP followed by KVX-053. Body weights are shown normalized to baseline (day 0). Mice were transitioned to the ethanol diet beginning on day 1, followed by intratracheal instillation of S1SP or vehicle on day 14. Animals were euthanized at the experimental endpoint on day 17. S1SP administration induced a significant reduction in body weight compared with vehicle-treated controls, whereas KVX-053 treatment attenuated S1SP-associated weight loss. Data are presented as mean ± SEM (n = 5 per group). Statistical significance was determined by two-way ANOVA with Tukey’s *post hoc* test. *p < 0.05 *versus* VEH; **p < 0.05 *versus* S1SP.

To assess the systemic impact of S1SP exposure and KVX-053 treatment in ethanol-fed mice, body weight was monitored daily throughout the experimental period (Figure 2). During the ethanol diet acclimation and maintenance phase, all groups exhibited comparable body weight trajectories, with no significant differences observed among vehicle-treated, S1SP-treated, or S1SP + KVX-053–treated animals. Following intratracheal instillation on day 14, mice receiving S1SP showed a progressive reduction in body weight compared to vehicle-treated controls. This weight loss became evident within 48–72 h after S1SP administration and persisted through the experimental endpoint. In contrast, treatment with KVX-053 significantly attenuated S1SP-induced body weight loss, with animals in the S1SP + KVX-053 group maintaining higher body weights than S1SP-only mice at the endpoint. These findings indicate that S1SP induces measurable systemic effects in ethanol-fed mice and that KVX-053 partially preserves body weight during acute lung injury.

### 3.2 S1SP induces bronchoalveolar inflammation, which KVX-053 mitigates

**FIGURE 3:**
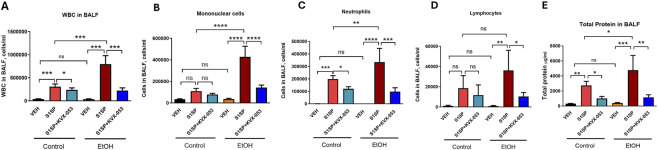
KVX-053 attenuates alcohol-exacerbated S1SP-induced lung inflammation. Inflammatory indices were measured in bronchoalveolar lavage fluid (BALF) collected 72 h after intratracheal administration of vehicle or SARS-CoV-2 Spike protein subunit 1 (S1SP) in K18-hACE2 mice maintained on control or ethanol (EtOH) diets. S1SP significantly increased BALF cellularity **(A–D)** and total protein content **(E)** in both diet groups, with a markedly greater response observed in ethanol-exposed mice. Treatment with the PTP4A3 inhibitor KVX-053 significantly reduced S1SP-induced inflammation, particularly in ethanol-exposed animals. Data are presented as mean ± SEM (n = 5 per group). Statistical significance was determined by one-way ANOVA with Tukey’s *post hoc* test. *p < 0.05, **p < 0.01, ***p < 0.001.

To determine whether KVX-053 attenuates alcohol-exacerbated S1SP-induced lung inflammation, inflammatory indices were assessed in BALF 72 h after intratracheal challenge (Figure 3). In ethanol-fed mice, S1SP administration resulted in a marked increase in BALF inflammatory parameters compared with vehicle-treated controls, indicating robust lung injury and inflammatory cell recruitment. Differential leukocyte analysis demonstrated that ethanol-exposed mice developed a predominantly mononuclear cell–associated inflammatory response following S1SP challenge, whereas control-diet animals exhibited a more neutrophil-predominant profile. Treatment with KVX-053 significantly reduced these S1SP-induced elevations, although values remained above vehicle levels. In control-diet animals, S1SP also increased BALF inflammatory indices, but the magnitude of the response was substantially lower than that observed in ethanol-fed mice. Collectively, these data demonstrate that chronic ethanol exposure amplifies S1SP-induced pulmonary inflammation and that KVX-053 effectively mitigates this alcohol-dependent inflammatory response.

### 3.3 KVX-053 suppresses alcohol-exacerbated S1SP-induced cytokine production

**FIGURE 4:**
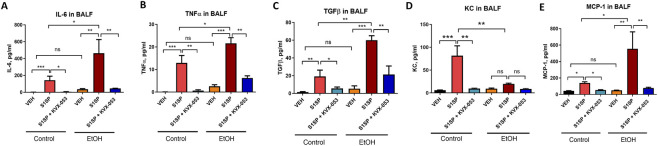
KVX-053 reduces alcohol-exacerbated S1SP-induced inflammatory mediator production in the lung. Concentrations of IL-6 **(A)**, TNF-α **(B)**, TGF-β **(C)**, KC/CXCL1 **(D)**, and MCP-1 **(E)** were measured in bronchoalveolar lavage fluid (BALF) collected 72 h after intratracheal instillation of vehicle or SARS-CoV-2 Spike protein subunit 1 (S1SP) in K18-hACE2 mice maintained on control or ethanol (EtOH) diets. S1SP increased BALF IL-6, TNF-α, TGF-β, and MCP-1 levels, with greater induction observed in ethanol-exposed mice. KC/CXCL1 levels were increased predominantly in control-diet mice and were not further enhanced by ethanol exposure. Treatment with the PTP4A3 inhibitor KVX-053 attenuated S1SP-induced inflammatory mediator production, particularly in ethanol-exposed animals. Data are presented as mean ± SEM (n = 4–5 per group). Statistical analysis was performed using one-way ANOVA followed by Tukey’s *post hoc* test. *p < 0.05, **p < 0.01, ***p < 0.001.

To evaluate the impact of KVX-053 on inflammatory mediator production, we measured cytokine and chemokine concentrations in BALF 72 h after intratracheal S1SP administration (Figure 4). In control-diet mice, S1SP increased BALF IL-6, TNF-α, TGF-β, and KC/CXCL1 levels, consistent with activation of an acute inflammatory response. In ethanol-exposed mice, S1SP induced a broader and more pronounced inflammatory profile, with marked increases in IL-6, TNF-α, TGF-β, and MCP-1. In contrast to the control-diet response, KC/CXCL1 was not further enhanced by ethanol exposure, suggesting a diet-dependent shift in chemokine pattern. Treatment with KVX-053 significantly attenuated S1SP-induced increases in IL-6, TNF-α, TGF-β, and MCP-1, with the most prominent effects observed in ethanol-exposed animals. These findings indicate that chronic ethanol consumption potentiates S1SP-induced inflammatory mediator production and that KVX-053 suppresses this exaggerated cytokine/chemokine response.

### 3.4 KVX-053 attenuates S1SP- and alcohol-induced activation of pro-inflammatory signaling pathways

**FIGURE 5:**
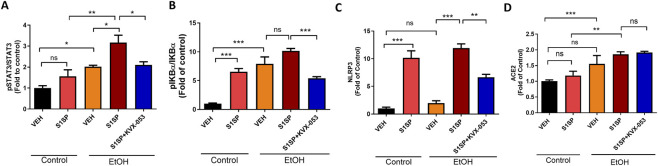
KVX-053 suppresses alcohol-exacerbated activation of STAT3, NF-κB, and NLRP3 signaling, but not ACE2 expression upregulation in the lung. Western blots densitometric analyses of lung tissue collected 72 h after intratracheal administration of vehicle or SARS-CoV-2 Spike protein subunit 1 (S1SP) in K18-hACE2 mice maintained on control or ethanol (EtOH) diets. Phosphorylated STAT3 (pSTAT3) to total STAT3 ratio **(A)**, phosphorylated IκBα (pIκBα) to total IκBα ratio **(B)**, NLRP3 **(C)** and ACE2 **(D)** protein levels were assessed. S1SP induced robust activation of STAT3 and NF-κB signaling and increased NLRP3 expression, with markedly greater responses observed in ethanol-fed mice. Treatment with the PTP4A3 inhibitor KVX-053 significantly attenuated these signaling responses, particularly under ethanol exposure, but did not affect the ethanol-induced upregulation of ACE2 expression. Densitometric values were normalized to total protein or β-actin and expressed relative to vehicle controls. Data are presented as mean ± SEM (n = 4–5 per group). Statistical analysis was performed using one-way ANOVA followed by Tukey’s *post hoc* test. *p < 0.05, **p < 0.01, ***p < 0.001.

To determine whether KVX-053 modulates key inflammatory signaling pathways activated by S1SP and chronic ethanol exposure, protein expression and phosphorylation status of key pro-inflammatory participants were assessed in lung tissue 72 h after intratracheal challenge (Figure 5). In ethanol-fed mice, S1SP administration resulted in a marked increase in STAT3 phosphorylation, enhanced degradation of IκBα consistent with NF-κB activation, and upregulation of NLRP3 inflammasome expression compared with vehicle-treated controls. These signaling responses were substantially greater in ethanol-exposed animals than in mice maintained on the control diet, indicating alcohol-dependent amplification of S1SP-induced inflammatory signaling. Treatment with KVX-053 significantly reduced STAT3 phosphorylation, preserved IκBα levels, and attenuated NLRP3 expression in ethanol-fed mice challenged with S1SP. In control-diet animals, S1SP also activated these pathways, but to a lesser extent, and KVX-053 produced a more modest suppressive effect. Together, these data demonstrate that KVX-053 effectively inhibits alcohol-exacerbated activation of STAT3, NF-κB, and inflammasome signaling pathways in S1SP-induced lung injury. In addition to inflammatory signaling pathways, lung expression of angiotensin-converting enzyme 2 (ACE2), the S1SP receptor, was examined (Figure 5D). Chronic ethanol feeding was associated with elevated ACE2 protein levels compared with control-diet animals. Following S1SP administration, ACE2 expression remained increased in ethanol-fed mice. Treatment with KVX-053 did not reduce ACE2 expression, indicating that PTP4A3 inhibition attenuates downstream inflammatory signaling without normalizing ethanol-associated ACE2 upregulation.

### 3.5 KVX-053 attenuates alcohol-exacerbated histologic lung injury induced by S1SP

**FIGURE 6:**
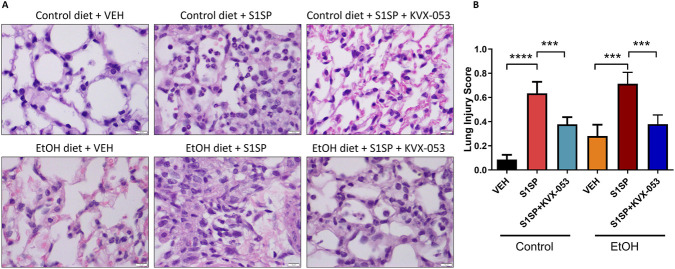
KVX-053 reduces alcohol-exacerbated histologic lung injury following S1SP challenge. Representative hematoxylin and eosin (H&E)–stained lung sections and corresponding lung injury scores obtained 72 h after intratracheal instillation of vehicle or SARS-CoV-2 Spike protein subunit 1 (S1SP) in K18-hACE2 mice maintained on control or ethanol (EtOH) diets **(A)**. Vehicle-treated control mice exhibit preserved alveolar architecture with minimal inflammatory infiltrates. S1SP induces prominent alveolar septal thickening, leukocyte accumulation, and architectural disruption, with more severe injury observed in ethanol-fed animals. Treatment with the PTP4A3 inhibitor KVX-053 attenuates histologic injury and reduces inflammatory cell infiltration. Scale bar = 10 μm. Lung injury scores **(B)** were determined by a blinded investigator using a standardized semi-quantitative scoring system. Data are presented as mean ± SEM (n = 4–5 per group). Statistical analysis was performed using one-way ANOVA followed by Tukey’s *post hoc* test. *p < 0.05, **p < 0.01, ***p < 0.001.

Representative hematoxylin and eosin (H&E)–stained lung sections and quantitative lung injury scores are shown in Figure 6. Vehicle-treated mice maintained on the control diet exhibited preserved alveolar architecture with thin septa and minimal inflammatory cell infiltration. In contrast, S1SP administration induced marked histologic injury characterized by alveolar septal thickening, increased interstitial and alveolar leukocyte accumulation, and focal disruption of airspace architecture. These changes were substantially more severe in ethanol-fed mice, consistent with alcohol-mediated amplification of lung injury. Qualitative assessment of inflammatory cell morphology revealed that S1SP-challenged control-diet mice exhibited predominantly polymorphonuclear leukocytes consistent with neutrophils, whereas ethanol-fed mice demonstrated a higher proportion of mononuclear cells consistent with monocytes/macrophages, consistent with our prior observations (Solopov et al., 2022a). Treatment with KVX-053 markedly reduced the severity of histologic abnormalities in both diet groups, with decreased cellular infiltration and partial restoration of alveolar structure. Quantitative lung injury scoring confirmed these observations, demonstrating significantly higher injury scores in S1SP-treated ethanol-fed mice compared with controls, and a significant reduction in injury severity following KVX-053 treatment.

### 3.6 KVX-053 attenuates S1SP- and alcohol-induced overexpression of PTP4A3

**FIGURE 7:**
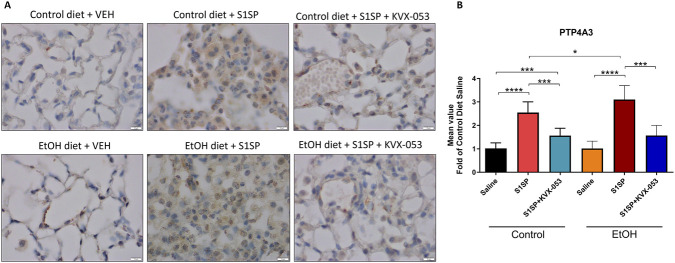
KVX-053 attenuates S1SP- and alcohol-induced overexpression of lung PTP4A3. Representative lung sections immunohistochemically stained for PTP4A3 **(A)** Brown chromogenic signal indicates PTP4A3 immunoreactivity; nuclei are counterstained with hematoxylin and eosin. S1SP exposure increased PTP4A3 staining within alveolar regions relative to saline controls, while ethanol feeding markedly amplified both staining intensity and distribution within inflamed alveolar parenchyma. KVX-053 treatment substantially reduced PTP4A3 signal and restricted staining to sparse focal regions. Scale bar = 10 μm. ImageJ-based quantification of PTP4A3 staining intensity/area from multiple high-power fields per lung **(B)** Data means SEM; *p < 0.05, ***p < 0.001, ****p < 0.0001; n = 5/.group.

Immunohistochemical analysis demonstrated marked differences in PTP4A3 expression across experimental conditions in both staining intensity and distribution within the alveolar compartment. In control lungs receiving saline, PTP4A3 immunoreactivity was minimal and limited to faint signals along alveolar walls. S1SP exposure under control diet conditions induced a moderate increase in PTP4A3 staining, principally along alveolar structures and within scattered intra-alveolar inflammatory infiltrates. Chronic ethanol feeding markedly amplified this response following S1SP challenge, producing more intense and diffuse cytoplasmic PTP4A3 staining throughout inflamed alveolar regions and dense cellular infiltrates, consistent with broader engagement of injured lung parenchyma. KVX-053 treatment substantially reduced PTP4A3 immunoreactivity in both control- and ethanol-fed S1SP-challenged mice, shifting the pattern toward sparse, focal staining and restoring overall distribution toward baseline alveolar architecture. These findings indicate that ethanol potentiates Spike-induced PTP4A3 activation *in vivo* and that pharmacologic PTP4A3 inhibition effectively suppresses this response at the tissue level (Figure 7).

## 4 Discussion

The present study demonstrates that pharmacologic inhibition of PTP4A3 with KVX-053 markedly attenuates lung injury caused by SARS-CoV-2 Spike protein subunit 1 (S1SP) in the context of chronic alcohol exposure. Using the K18-hACE2 model, we show that ethanol feeding amplifies S1SP-induced pulmonary inflammation, vascular permeability, cytokine production, and activation of STAT3, NF-κB, and NLRP3 inflammasome signaling. KVX-053 significantly suppressed each of these alcohol-exacerbated endpoints, including lung architecture damage, while preserving body weight and improving barrier integrity. These findings extend our prior work (Solopov et al., 2022a) demonstrating alcohol-dependent sensitization to Spike-mediated lung injury and establish PTP4A3 inhibition as an effective strategy to blunt this pathological amplification. Importantly, while prior studies have characterized S1SP-induced lung injury and alcohol-mediated amplification independently, the present study demonstrates that PTP4A3 inhibition with KVX-053 attenuates the altered inflammatory signaling that emerges specifically in the alcohol-conditioned lung.

Consistent with emerging evidence that Spike protein alone can drive inflammatory and vascular injury independently of viral replication, S1SP exposure in this model elicited robust cytokine induction, leukocyte recruitment, and signaling pathway activation. Prior studies have shown that recombinant Spike or S1 fragments disrupt endothelial barrier function, activate Toll-like receptor signaling, and promote inflammatory gene expression in the absence of infectious virus (Buzhdygan et al., 2020; Lei et al., 2021; Shirato and Kizaki, 2021). Our previous work similarly demonstrated that intratracheal S1SP administration reproduces key features of COVID-19–associated lung injury, including STAT3 and NF-κB activation, alveolar protein leak, and inflammatory cell infiltration (Colunga Biancatelli et al., 2021). The present findings reinforce the concept that Spike-driven host inflammatory responses constitute a major pathogenic component of acute lung injury and provide a tractable platform for evaluating host-directed therapeutic interventions.

Alcohol exposure profoundly altered the magnitude and quality of the pulmonary response to S1SP. Ethanol-fed mice exhibited significantly higher bronchoalveolar leukocyte counts, greater protein leakage, elevated cytokine concentrations, and more severe histologic injury compared with control-diet animals, reproducing the finding observed in our previous study (Solopov et al., 2022a). Chronic alcohol exposure disrupts epithelial and endothelial barrier integrity in the lung by altering tight junction organization and cytoskeletal stability, leading to increased permeability and impaired barrier function (Mehta and Guidot, 2017; Guidot et al., 2000). Alcohol also impairs innate immune regulation, including reduced alveolar macrophage phagocytic capacity, altered cytokine signaling, and diminished mucociliary clearance, thereby compromising pulmonary host defense (Simet and Sisson, 2015; Happel and Nelson, 2005). In parallel, ethanol increases oxidative stress in the lung, making pulmonary tissues more vulnerable to secondary inflammatory challenges and more prone to excessive injury during infectious or sterile inflammation (Brown et al., 2001; Yeligar et al., 2012). Clinically, AUD is associated with increased risk of pneumonia, sepsis, and ARDS, and recent epidemiologic studies suggest worse outcomes following SARS-CoV-2 infection among individuals with unhealthy alcohol use (Bhalla et al., 2021; Bailey K. L. et al., 2022). Our current data provides mechanistic support for these clinical observations by demonstrating that alcohol directly amplifies Spike-triggered inflammatory signaling at the tissue level.

An additional observation in this study was a shift in inflammatory cell composition between diet groups. In control-fed mice, S1SP-induced infiltrates were predominantly neutrophilic, consistent with an acute innate inflammatory response. In contrast, ethanol-exposed animals exhibited a higher proportion of mononuclear cells consistent with monocytes/macrophages, reproducing patterns observed in our prior work (Solopov et al., 2022a). In the present study, this shift in cellular composition was paralleled by differences in cytokine and chemokine profiles. Specifically, S1SP exposure in control-diet mice was associated with increased KC/CXCL1, consistent with neutrophil recruitment, whereas ethanol-exposed mice demonstrated a distinct pattern characterized by marked increases in MCP-1 along with IL-6, TNF-α, and TGF-β, with relatively lower KC induction. This altered leukocyte profile may reflect alcohol-induced changes in chemokine gradients, macrophage recruitment, monocyte differentiation, or impaired neutrophil clearance. Such shifts could influence not only the magnitude of inflammation but also its resolution kinetics and downstream remodeling responses. Other experimental and clinical studies also have shown that chronic alcohol exposure alters pulmonary chemokine gradients and macrophage trafficking, favoring monocyte recruitment while impairing effective neutrophil function and clearance (Simet and Sisson, 2015; Happel and Nelson, 2005). Alcohol has also been shown to impair macrophage phagocytic capacity and macrophage clearance of apoptotic inflammatory cells (efferocytosis), which can prolong inflammation and delay resolution of lung injury (Mehta and Guidot, 2017; Yeligar et al., 2012). In models of acute lung injury, sustained monocyte/macrophage accumulation is associated with prolonged cytokine production and increased risk of fibroproliferative remodeling (Grommes and Soehnlein, 2011; Herold et al., 2011). Accordingly, alcohol-driven shifts in leukocyte composition may influence not only the severity of acute injury but also subsequent repair processes. Given the altered inflammatory landscape observed in ethanol-exposed lungs, it is particularly important to determine whether KVX-053 can effectively modulate this dysregulated signaling environment.

KVX-053 (JMS-053) has been previously characterized as a potent, reversible, noncompetitive inhibitor of PTP4A3 (McQuee et al., 2018). Kinetic analyses demonstrated that JMS-053 does not compete directly with substrate at the catalytic site, supporting a mechanism consistent with allosteric modulation. This distinction is important because protein tyrosine phosphatases, including the PRL family, have historically been considered challenging drug targets due to the conserved nature of their catalytic domains and limited success of early orthosteric inhibitors (Lazo et al., 2017; Xiao et al., 2024). Functional studies showed that inhibition of PTP4A3 by KVX-053 suppresses downstream signaling pathways and cellular phenotypes associated with PTP4A3 activity. In the present study, the protective effects of KVX-053 in alcohol-exacerbated S1SP-induced lung injury are therefore consistent with selective attenuation of PTP4A3–dependent inflammatory amplification. Mechanistically, KVX-053 consistently suppressed activation of STAT3 and NF-κB pathways and reduced NLRP3 inflammasome expression in ethanol-exposed lungs following S1SP challenge. These pathways represent key mechanisms linking Spike-mediated signaling with alcohol-induced immune dysregulation. PTP4A3 regulates multiple signaling pathways linked to inflammatory activation. The differential activation of signaling pathways observed in this study suggests that NF-κB–associated signaling may represent the dominant mechanism driving cytokine production in response to S1SP alone, whereas STAT3 activation becomes more prominent in the context of chronic ethanol exposure. This may reflect alcohol-induced priming of inflammatory pathways, as chronic ethanol exposure has been shown to disrupt pulmonary barrier integrity, increase oxidative stress, and alter inflammatory signaling and macrophage function in the lung (Guidot and Hart, 2005; Joshi and Guidot, 2007; Moss and Burnham, 2006). S1SP exposure in control-diet animals produced a modest increase in STAT3 activation; however, this effect did not reach statistical significance under the present experimental conditions. In contrast, ethanol exposure was associated with a more robust increase in STAT3 signaling, suggesting that chronic alcohol exposure potentiates STAT3 pathway activation in response to S1SP. In several cellular models, PTP4A3 overexpression promotes persistent STAT3 phosphorylation and increases STAT3-dependent transcription (Rubio and Köhn, 2016; Slørdahl et al., 2016; Lazo et al., 2023). Conversely, pharmacologic inhibition of PTP4A3 suppresses STAT3 activation and downstream gene expression (Colunga Biancatelli et al., 2022). PTP4A3 has also been shown to activate NF-κB signaling through interaction with the small GTPase RAP1 and enhanced phosphorylation and nuclear accumulation of the p65 NF-κB subunit (Lian et al., 2013). Together, these studies support a role for PTP4A3 as an upstream regulator of inflammatory signaling pathways relevant to barrier dysfunction and tissue injury. Consistent with these signaling effects, PTP4A/PRL inhibition has been shown to stabilize endothelial barrier function in cellular permeability models and to prevent/repair pulmonary endothelial hyperpermeability while reducing acute lung injury severity *in vivo* in Spike S1–driven K18-hACE2 models (Colunga Biancatelli et al., 2022; McQuee et al., 2018). These findings are consistent with broader evidence that preservation of pulmonary endothelial barrier integrity represents an important therapeutic strategy in inflammatory lung injury (Barabutis et al., 2020).

Our findings align with prior studies demonstrating that KVX-053 protects against Spike-induced acute lung injury, ventilator-induced lung injury, and endothelial barrier disruption *in vitro* and *in vivo* (Colunga Biancatelli et al., 2021; Solopov et al., 2024; Colunga Biancatelli et al., 2025b). Importantly, the present data extends these protective effects to a clinically relevant comorbidity context, namely, chronic alcohol exposure. Immunohistochemical analysis further supports a mechanistic role for PTP4A3 in Spike-driven lung injury, consistent with our prior observation of increased pulmonary PTP4A3 expression in the S1SP model (Solopov et al., 2024). In the present study, S1SP exposure increased PTP4A3 immunoreactivity within alveolar structures, and this response was markedly amplified by chronic ethanol feeding, producing broader and more intense staining within inflamed alveolar compartments. Importantly, KVX-053 substantially attenuated PTP4A3 expression under both control and ethanol-sensitized conditions and restored a sparse distribution approaching baseline. Several immune-modulating agents, including JAK inhibitors, have demonstrated protective effects in SARS-CoV-2–associated inflammatory lung injury and COVID-19 by suppressing cytokine-driven inflammatory signaling pathways (Kalil et al., 2021; Stebbing et al., 2020). In contrast, KVX-053 acts upstream by inhibiting PTP4A3, thereby modulating both NF-κB and STAT3 signaling pathways. Interestingly, ACE2 expression remained elevated in ethanol-fed mice despite KVX-053 treatment, indicating that PTP4A3 inhibition primarily modulates downstream inflammatory signaling rather than upstream receptor expression. Several studies including our prior work, have reported that chronic ethanol exposure increases ACE2 expression in pulmonary and extrapulmonary tissues, potentially through oxidative stress–dependent and epigenetic regulatory mechanisms (Solopov et al., 2022a; Leung et al., 2020; Nowak et al., 2020). Alcohol-associated ACE2 upregulation has been proposed to increase cellular susceptibility to SARS-CoV-2 binding and may enhance sensitivity to Spike-mediated signaling even in the absence of productive viral infection (Friske et al., 2023). The persistence of elevated ACE2 despite suppression of inflammatory signaling in the present study suggests that alcohol-driven receptor regulation may operate independently of downstream pathway normalization and may remain a risk modifier even when inflammatory amplification is therapeutically controlled. Importantly, the ability of KVX-053 to attenuate lung injury despite sustained ACE2 elevation underscores the central role of intracellular signaling cascades, rather than receptor abundance alone, in determining injury severity. Targeting downstream inflammatory amplification pathways, primarily through modulation of NF-κB–associated inflammatory signaling, with additional contributions from STAT3 pathways, particularly under ethanol exposure, may therefore provide therapeutic benefit even when upstream susceptibility factors such as ACE2 expression cannot be readily modified.

## Data Availability

The original contributions presented in the study are included in the article/supplementary material, further inquiries can be directed to the corresponding author.
